# Ventilating Chambers

**Published:** 1861-02

**Authors:** 


**VENTILATING CHAMBERS.**—When it is considered that pure air is essential to the purification of the blood, and that the food we eat never becomes nutriment until it meets with the air in the lungs, and when it is furthermore remembered that a full third of our entire existence is passed in our sleeping apartments, it must be clear to the commonest understanding that the difference between breathing a pure and impure air while we are asleep is literally incalculable as to the effects upon our happiness and well-being. How an impure air is caused and how it may be avoided are plainly treated of in our new book on SLEEP, including, as it does, the general subjects of sleeping, ventilation, the planning and warming of houses, etc., etc.

